# Musical imagery depends upon coordination of auditory and sensorimotor brain activity

**DOI:** 10.1038/s41598-019-53260-9

**Published:** 2019-11-14

**Authors:** Rebecca W. Gelding, William F. Thompson, Blake W. Johnson

**Affiliations:** 1 Department of Cognitive Science, Macquarie University, Sydney, NSW 2109 Australia; 2 Department of Psychology, Macquarie University, Sydney, NSW 2109 Australia

**Keywords:** Perception, Human behaviour

## Abstract

Recent magnetoencephalography (MEG) studies have established that sensorimotor brain rhythms are strongly modulated during mental imagery of musical *beat* and *rhythm*, suggesting that motor regions of the brain are important for temporal aspects of musical imagery. The present study examined whether these rhythms also play a role in non-temporal aspects of musical imagery including musical *pitch*. Brain function was measured with MEG from 19 healthy adults while they performed a validated musical pitch imagery task and two non-imagery control tasks with identical temporal characteristics. A 4-dipole source model probed activity in bilateral auditory and sensorimotor cortices. Significantly greater β-band modulation was found during imagery compared to control tasks of auditory perception and mental arithmetic. Imagery-induced β-modulation showed no significant differences between auditory and sensorimotor regions, which may reflect a tightly coordinated mode of communication between these areas. Directed connectivity analysis in the θ-band revealed that the left sensorimotor region drove left auditory region during imagery onset. These results add to the growing evidence that motor regions of the brain are involved in the top-down generation of musical imagery, and that imagery-like processes may be involved in musical perception.

## Introduction

The silent generation of music in one’s own mind is a common human experience. Musical imagery is well developed in musicians: Mozart reportedly experienced his compositions through polyphonic imagery^1^, while Beethoven was (presumably) forced rely on musical imagery to compose his late symphonies, a period when he was largely or completely deaf^2^. The pianist Glenn Gould had unimpaired hearing but preferred to study music by reading it rather than playing it, indicating that musical imagery can be a powerful strategy in and of itself, and not merely a backup strategy necessitated by deafness^3^. Among contemporary composers, functional magnetic resonance imaging (fMRI) measurements show that Sting uses highly similar brain regions when listening to or imagining music^4^. However, musical imagery is not restricted to the musical elite; the mental replaying of music is an everyday experience for non-musicians, and involuntary musical imagery or “earworms” is common^5,6^.

Imagery and perception are thought to use overlapping neural mechanisms^7^, and auditory brain regions, particularly in the right hemisphere, are active during musical imagery^8–10^. Recent work has also pointed to an important role for motor regions of the brain in auditory perception and imagery. Several fMRI studies have reported increased BOLD responses in motor areas during musical imagery including primary motor, pre-motor, parietal and inferior frontal cortex^11–14^. Supplementary motor area (SMA) and pre-SMA become active both during motor sequence learning and during anticipation of sound sequences, suggesting the use of motoric predictive mechanisms in both domains^15^. There is also evidence that individual differences in vividness of auditory imagery are correlated with grey matter volume in the SMA, parietal and prefrontal regions, suggesting that the generation of auditory images requires access to auditory-motor representations^16^. Moore^17^ and Schaefer^18^ have suggested that this overlap in imagery and perception may reflect a type of “constructive imagery”, invoking temporal prediction as an aid to auditory perception. Taken together, these lines of evidence suggest that music perception and imagery require the coordination of both auditory and motor regions of the brain, even when no overt actions are required. Such findings are consistent with current neurophysiological frameworks that posit ventral and dorsal streams for processing auditory information comparable to those of the visual system^19–21^.

Beta-band (β: ~13–30 Hz) and mu-band (μ: ~8–12 Hz) oscillations provide robust neurophysiological markers of motor cortical function that can be measured noninvasively with MEG^22,23^. Recent work has implicated the β-rhythm in temporal predictions of sounds and sound sequences^24–28^. Beta-band responses to sounds show a characteristic event related desynchronisation (ERD) at a latency of about 200 ms after sound onset, followed by a rebound event related synchronisation (ERS). Fujioka *et al*.^26^ found that the slope of the β-ERS peaks just before an expected tone. The β-ERD also varies as a function of a listener’s metrical interpretation of a simple rhythmic pattern^27^. Recent evidence has also confirmed that β-band modulation reflects predictability of pitch (that is ‘what’) and not just timing (or ‘when’ an event will occur), since greater trial-by-trial ERD prior to a predictable tone is related to a reduced P3a amplitude after the tone^29,30^. Finally, both physical and imagined accents on a downbeat modulate the β-band response, suggesting that the β-band plays a role in the temporal coordination of auditory and motor operations in music perception and imagery^25,27^. Oscillations in the μ-band have been found to increase in parieto-occipital regions more in imagery than perception^31^, but decrease in bilateral auditory and right precentral regions during imagery of familiar song occurring white noise^32^. Better auditory working memory ability is associated with greater ERD across 8–18 Hz^33^. Thus, converging evidence suggests that β-band oscillations, and possibly also μ-band oscillations, are functionally associated with the temporal and spectral features of music perception, as well as the temporal features of musical imagery including rhythm, accent and beat.

Recent approaches using magnetoencephalography (MEG) have isolated the primary auditory and sensorimotor regions to investigate how they coordinate in perceptual activities, specifically in beat perception^34^, temporal prediction^35^ and passive listening^36^. Results from these studies suggest the right auditory cortex is primarily involved in beat perception^34^. Using directed Phase Transfer Entropy (dPTE) connectivity analysis for measuring the direction of communication between regions at different frequency bands, Morillon and Baillet^35^ showed that in the β-band (18–24 Hz), the bilateral sensorimotor regions drive activity in the auditory cortex during temporal prediction, whilst in lower frequencies (2–4 Hz) the auditory regions drive the sensorimotor regions. Enhanced functional connectivity in the θ-band between the left sensorimotor and the bilateral auditory regions has also been found during passive listening to an unfamiliar instrument, after short term motor training in the action required to play the instrument^36^. Theta-band connectivity is thought to reflect long range communication between brain regions^37^, and brain stimulation of the left intraparietal sulcus at the theta frequency has also been shown to improve performance of imagery manipulation (mental reversal of melody) but not maintenance of melodies^38^. Given the overlapping activation in these areas during perception and imagery, investigating the coordination and connectivity of the primary auditory and sensorimotor regions in the θ, μ and β frequencies could prove to be fruitful in the exploration of similarities and differences between musical imagery and perception.

Hence the aim of the present study was to investigate oscillatory activity in bilateral auditory and sensorimotor regions during musical imagery. Two separate conditions with similar visual and temporal attributes were used: perception condition – identical to imagery but with all tones sounded – and mental arithmetic – a control for working memory. In the imagery task, participants were prompted with visual cues of up and down arrows to silently imagine successive changes in the pitch of a piano note up and down the major scale. Performance on this imagery task was then objectively measured by requiring participants to decide if a probe note did or did not match to the result of the specified manipulations.

Our experiment was designed to address three main questions: (1) Does musical imagery differentially modulate β-band oscillations relative to other mental operations (music perception, and mental arithmetic) with identical temporal features? (2) Are β-band oscillations differentially modulated by musical imagery in different brain regions (sensorimotor versus auditory) or hemispheres (right versus left)? (3) Do musical imagery and perception differ in how they direct functional connections between bilateral sensorimotor and auditory regions? We hypothesised that (1) musical imagery would require greater coordination between auditory and sensorimotor regions compared to perception and mental arithmetic, and therefore would show greater β-band modulation^26^; (2) greater modulation would be seen in the right than the left hemisphere^39^; (3) there would be greater sensorimotor to auditory directed connectivity in musical imagery than perception^35^.

## Results

**Figure 1:**
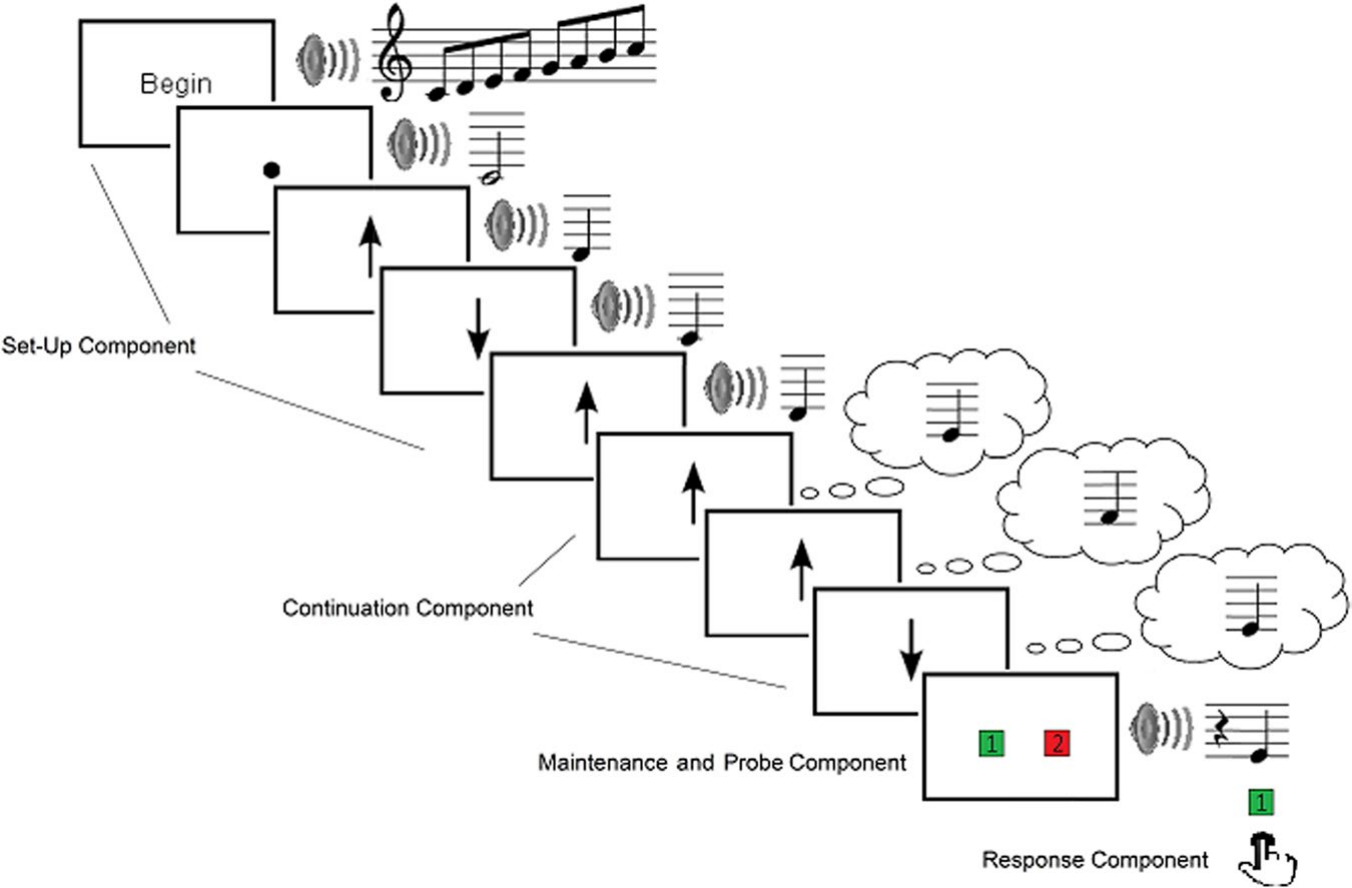
Schematic of a MEG imagery trial. Arrow cues were presented at a constant rate of 1 per second. In this example the accurate response is “Correct” which is indicated by pressing the green button.

**Figure 2:**
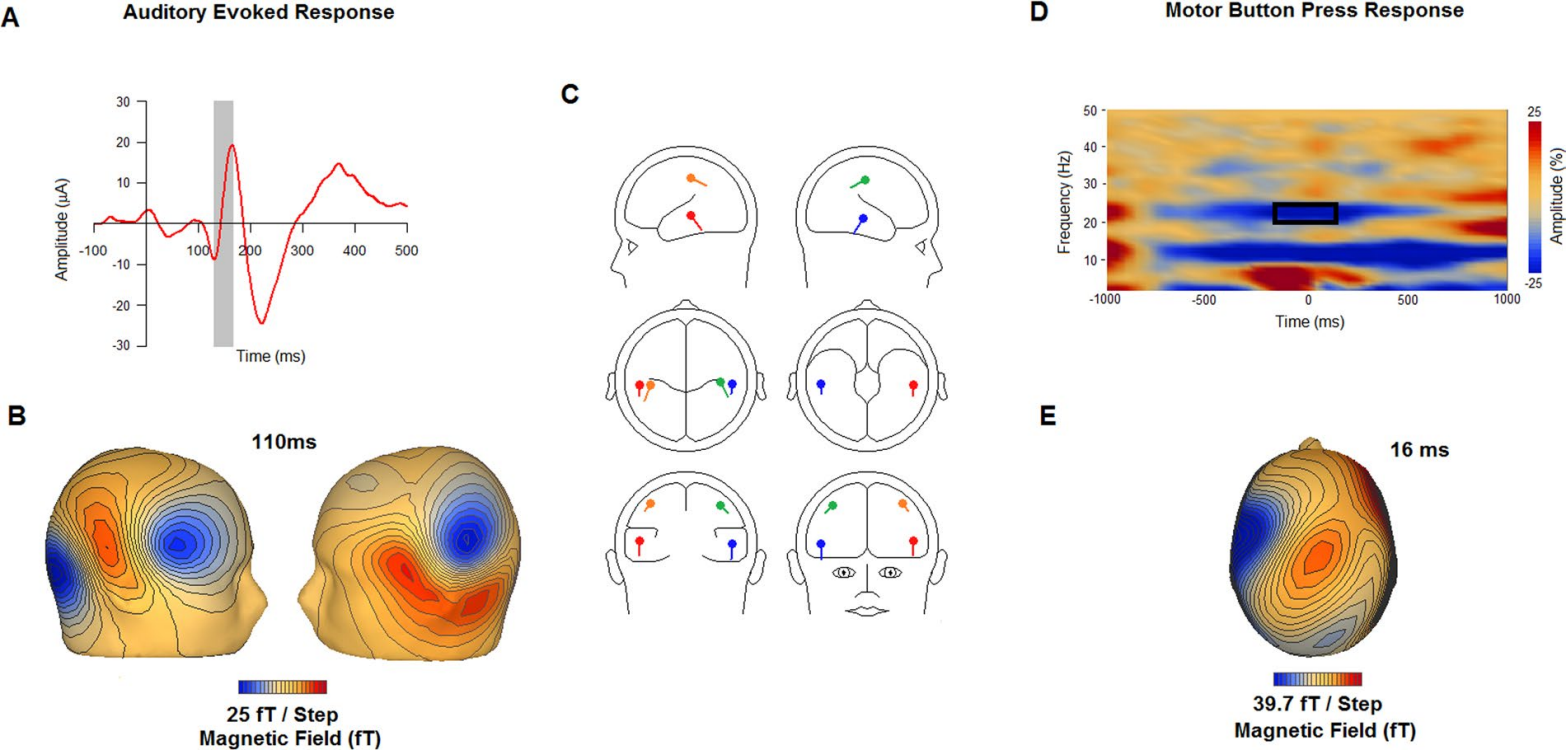
(**A**) Auditory source waveform from single participant with shaded region indicating time window used to fit the auditory dipoles. (**B**) Surface topography of auditory evoked response. (**C**) Mean auditory and sensorimotor sources from all participants. (**D**) Time frequency plot for single participant at central sensor for right-hand button press. (**E**) Surface topography of motor evoked response.

**Figure 3:**
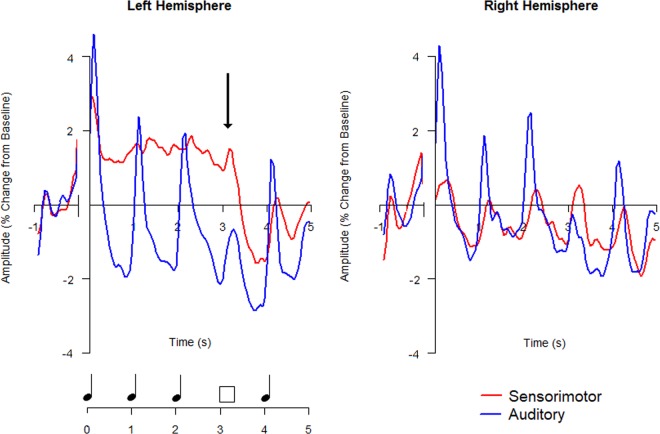
3–10 Hz frequency band activity (reflecting the evoked response) from bilateral auditory and sensorimotor sources during Perception trials, for all participants. Arrow on left hemisphere plot shows start of the readiness field of sensorimotor cortex in response to the pre-probe screen.

Participants completed a validated musical imagery task, the Pitch Imagery Arrow Task (PIAT, Fig. 1) and two control conditions with identical temporal attributes: pitch perception and mental arithmetic. Individual differences were measured for musical experience (MEI) and auditory imagery vividness (BAIS). (*Methods*). Individually fitted 4-dipole source models probed activity in bilateral auditory and sensorimotor cortices (Fig. 2). Figure 3 illustrates the performance of the source model using stimulus-locked activity and response-locked activity from Perception trials: auditory sources are clearly maximally sensitive to sound onset activity, while the left sensorimotor source is maximally sensitive to the response-locked readiness field (right hand button press). These results confirm that the model sources are differentially sensitive to the activities expected in the modelled brain regions.

## Behavioural Results

Mean accuracy on the Imagery task was 80.2% (SD = 14.9%), significantly lower than performance on Perception (M = 98.4%; SD = 3%) and Maths (M = 91.9%; SD = 8%) conditions (*F*_(2,54)_ = 16.18; *p* < 0.001; η^2^ = 0.037), reflecting greater difficulty of the Imagery task. Mean hit reaction time (ms) was significantly slower for Imagery trials (M = 834, SD = 169) than for Perception (M = 707, SD = 96) or Maths trials (M = 685, SD = 113) (*F*_(2,54)_ = 7.36; *p* = 0.002; η^2^ = 0.22).

Accuracy on the Imagery and Maths tasks was significantly correlated with musical experience index (MEI; *r* *=* 0.50, *p* *=* 0.028; and *r* *=* 0.47, *p* *=* 0.04, respectively). In contrast to the results of Gelding *et al*.^40^, accuracy on the Perception task was not significantly related to MEI, due to a ceiling effect in which all but 3 participants obtained 100% accuracy on the Perception trials.

Debrief vividness ratings (a 1–5 self-rated score of vividness experienced during task completion obtained at the end of the experiment) were significantly positively correlated with Imagery accuracy (*r* *=* 0.071, *p* *<* 0.001) and MEI (*r* = 0.504, *p* = 0.028), and negatively correlated with Imagery reaction time (*r* = −0.69, *p* *=* 0.001) and Perception reaction time (*r* = −0.536, *p* *=* 0.018).

### Bucknell auditory imagery scale

Imagery accuracy (percent correct) was positively correlated with both the vividness (BAIS-V) (*r* = 0.52, *p =* 0.023) and control (BAIS-C) (*r =* 0.48, *p =* 0.037) subscales of the BAIS, consistent with the results of the previous behavioural study^40^. Neither subscale showed a significant correlation with accuracy in the Perception or Maths condition. The BAIS-C subscale showed a significant negative correlation with mean hit reaction times in both the Imagery (*r = *−0.54*, p =* 0.017) and Perception (*r = *−0.56*, p =* 0.014) conditions.

Taken together, the behavioural results provide strong support for our contention that participants performed the Imagery task with a pitch imagery strategy: imagery performance was gauged with an objective behavioural measure; performance was significantly positively correlated with an independent measure of individual imagery ability (BAIS); and all participants confirmed the use of a musical imagery to complete the task (i.e. hearing the sounds in their head or singing them in their head) in post-experimental debriefings.

## MEG Results

### Normalised β-band time course

**Figure 4:**
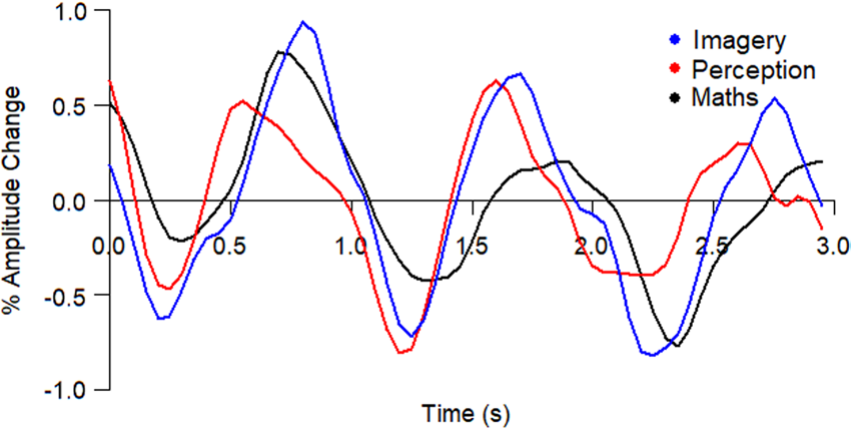
Mean normalised β-band time course for Imagery, Perception and Maths Condition during the last 3 arrows of each trial (arrow onset at t = 0, 1, 2 s).

The mean induced β-band time course over 0–3 seconds, corresponding to the last 3 arrows presented in each condition, were calculated, normalised and plotted (Fig. 4). The absolute area under the (rectified) curve from this figure showed greater mean modulation for Imagery (1.168%) than Perception (0.89%) or Maths (0.843%).

### Power of 1 Hz modulation

Given the isochronous presentation of the arrows at 1 per second in all conditions, the 1 Hz power of the average time courses in three oscillatory bands of interest (evoked response (3–10 Hz), induced μ-band (8–12 Hz) and induced β-band (15–25 Hz)) for each condition and source were calculated and compared.

### Evoked response (3–10 Hz)

**Figure 5:**
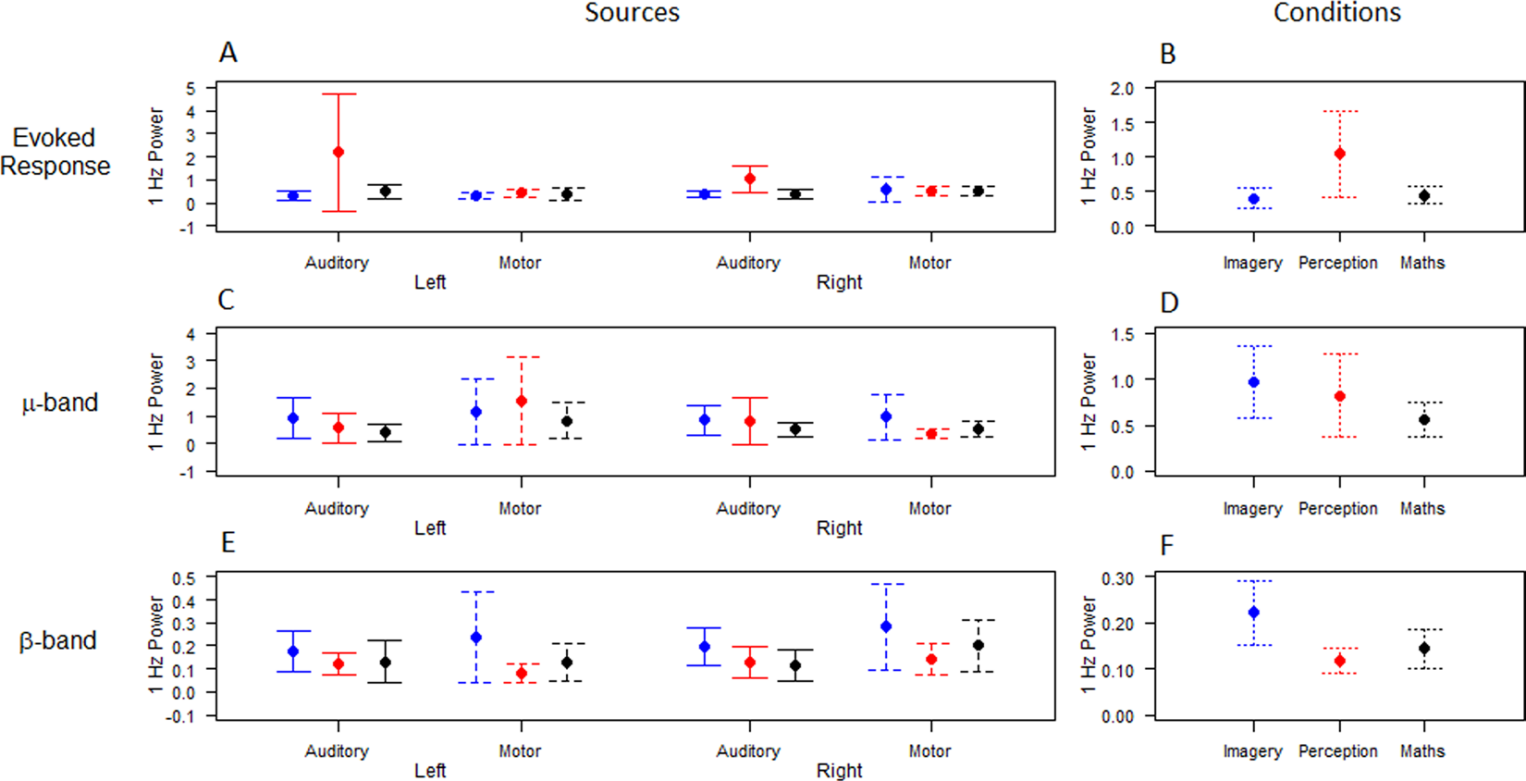
1 Hz Power during continuation period for all three conditions. (**A,B)** Evoked Response (3–10 Hz); (**C,D**) μ-band (8–12 Hz) and (**E,F**) β-band (15–25 Hz). 1^st^ Column shows mean results and 95% confidence intervals for each of the four sources for three conditions (Imagery: blue; Perception: red; Maths: black), with auditory sources having solid lines and sensorimotor sources having dashed lines (**A,C,E**). 2^nd^ Column shows mean results and 95% confidence intervals of all sources for each of the three conditions (**B,D,F**).

Repeated measures ANOVAs (3 Conditions (Imagery; Perception; Maths) x 2 Hemispheres (Right; Left) computed for 1 Hz power over time window of 0–2.95 s were calculated for Auditory and Sensorimotor sources separately. Greenhouse-Geisser corrections were applied where appropriate. No significant main effects or interactions were found. Figure 5A confirms substantially greater 1 Hz power in the Auditory sources in the Perception condition relative to the Imagery and Maths conditions, however correcting for sphericity revealed these differences were not significant.

### μ-Band (8–12 Hz)

The 3 × 2 repeated measures ANOVAs for Auditory and Sensorimotor sources also showed no significant main effects or interactions. This is seen in Fig. 5D.

### β-Band (15–25 Hz)

The 3 × 2 repeated measures ANOVA for the induced β-band 1 Hz power from Motor sources showed a significant main effect of Condition after correcting for sphericity using Greenhouse-Geisser (*F*_(2,108)_ = 3.788, *p* = 0.0045, GG ε = 0.770). Post hoc comparisons (Tukey HSD, α = 0.05) showed that Imagery power was significantly greater than Perception (*p* = 0.0042), and there was no significant difference between Perception and Maths (*p* = 0.0635) or Imagery and Maths (*p* = 0.0279). The 3 × 2 repeated measures ANOVA for the induced β-band 1 Hz power from Auditory sources also showed a significant main effect of Condition, however after correcting for sphericity using Greenhouse-Geisser this was no longer significant at *p* < 0.005 (*F*_(2,108)_ = 3.444, *p* = 0.0051, GG ε = 0.857). This suggests that β-band magnitude was modulated significantly more during auditory Imagery than during auditory Perception, as seen in Fig. 5F, particularly in the Sensorimotor sources.

## Exploratory Analyses

### β-Band

Additional analyses explored the sensitivity and power of additional metrics of β-power modulation: timing, and magnitude of Maximum β-ERD (Max ERD_TIME_ and Max ERD_AMP_, respectively) and Maximum β-ERS (Max ERS_TIME_ and Max ERS_AMP_, respectively), and the difference between them (Time Difference and Magnitude Difference) (*Method*). All ANOVAs were corrected for Sphericity where Mauchly’s test was significant, as well as False Discovery Rate.

### Timing of β-ERD

A significant main effect of Condition was found for Max ERD_TIME_ (*F*_(2,216)_ = 20.26, *p* = 0.0001, GG ε = 0.866, FDR corrected). Post hoc Tukey HSD comparisons revealed that the Max ERD_TIME_ in Perception (M = 225.23 ms, SD = 61.19 ms) occurred significantly earlier than both Imagery (M = 262.06 ms, SD = 71.68 ms) (*p* = 0.003) and Maths (M = 310.96 ms, SD = 71.42 ms) (*p* < 0.0001), and Imagery was earlier than Maths (*p* < 0.0001).

### Timing of β-ERS

**Figure 6:**
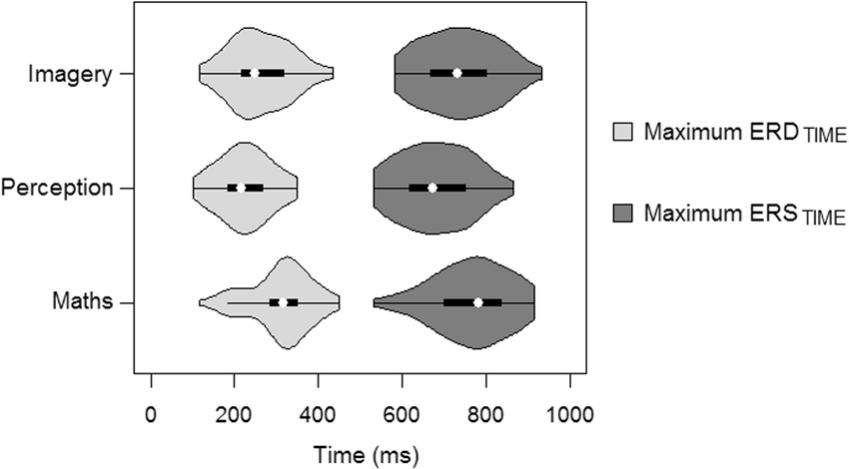
β-band: Max ERD_TIME_ (light grey) and Max ERS_TIME_ (dark grey) for all conditions, averaged across all sources. Perception condition was significantly earlier for Max ERD_TIME_ and Max ERS_TIME_ than Imagery or Maths. Imagery condition was faster than Maths for Max ERD_TIME_ but no difference for Max ERS_TIME._

A significant main effect of Condition was also found for Max ERS_TIME_ (*F*_(2,216)_ = 12.51, *p =* 0.003, GG ε = 0.839, FDR corrected). Post hoc Tukey HSD comparisons showed that Max ERS_TIME_ in Perception (M = 679.83 ms, SD = 83.86 ms) occurred again significantly earlier than both Imagery (M = 736.62 ms, SD = 87.01 ms) (*p* = 0.0002) and Maths (M = 766.67 ms, SD = 87.73 ms) (*p* < 0.0001), but there was no significant difference in the timing of the rebound for Imagery and Maths (*p* = 0.0083). Figure 6 shows a summary of these main results for Max ERD_TIME_ and Max ERS_TIME_, with Perception β-ERD and β-ERS occurring earlier than the other conditions.

### Magnitude difference

A main effect of Condition was also found in Magnitude Difference (*F*_(2,216)_ = 6.43, *p* = 0.0338, FDR corrected), that is the difference between the maximum ERS and maximum ERD. Post hoc Tukey HSD comparisons showed that Imagery (M = 2.6%, SD = 1.2%) had a significantly larger Magnitude Difference (i.e. greater difference between Max ERS_AMP_ and Max ERD_AMP_) than Perception (M = 2.2%, SD = 0.75%) (*p* = 0.029) and Maths (M = 2.2%, SD = 1.1%) (*p* = 0.019). There was no difference between Perception and Maths.

These results corroborate the 1 Hz Power analysis and suggest that β-band magnitude was more significantly modulated during Imagery than Perception. Moreover, given β-band modulation, as measured by the Magnitude Difference metric, was greater for Imagery than Maths, the modulation appears to be specific to pitch imagery, and less evident during silent mathematical computations. No other significant main effects were found in Time Difference or Magnitude Difference.

In addition, to validate the use of the 1 Hz Power FFT as a measure of modulation, the correlation between Magnitude Difference and 1 Hz Power in β-band was calculated, and found to be significant (*r* = 0.811, *p* < 0.0001). This finding confirms that both metrics provide similar information about the modulation of oscillations occurring during the last three arrows across conditions.

### Connectivity analysis

**Figure 7:**
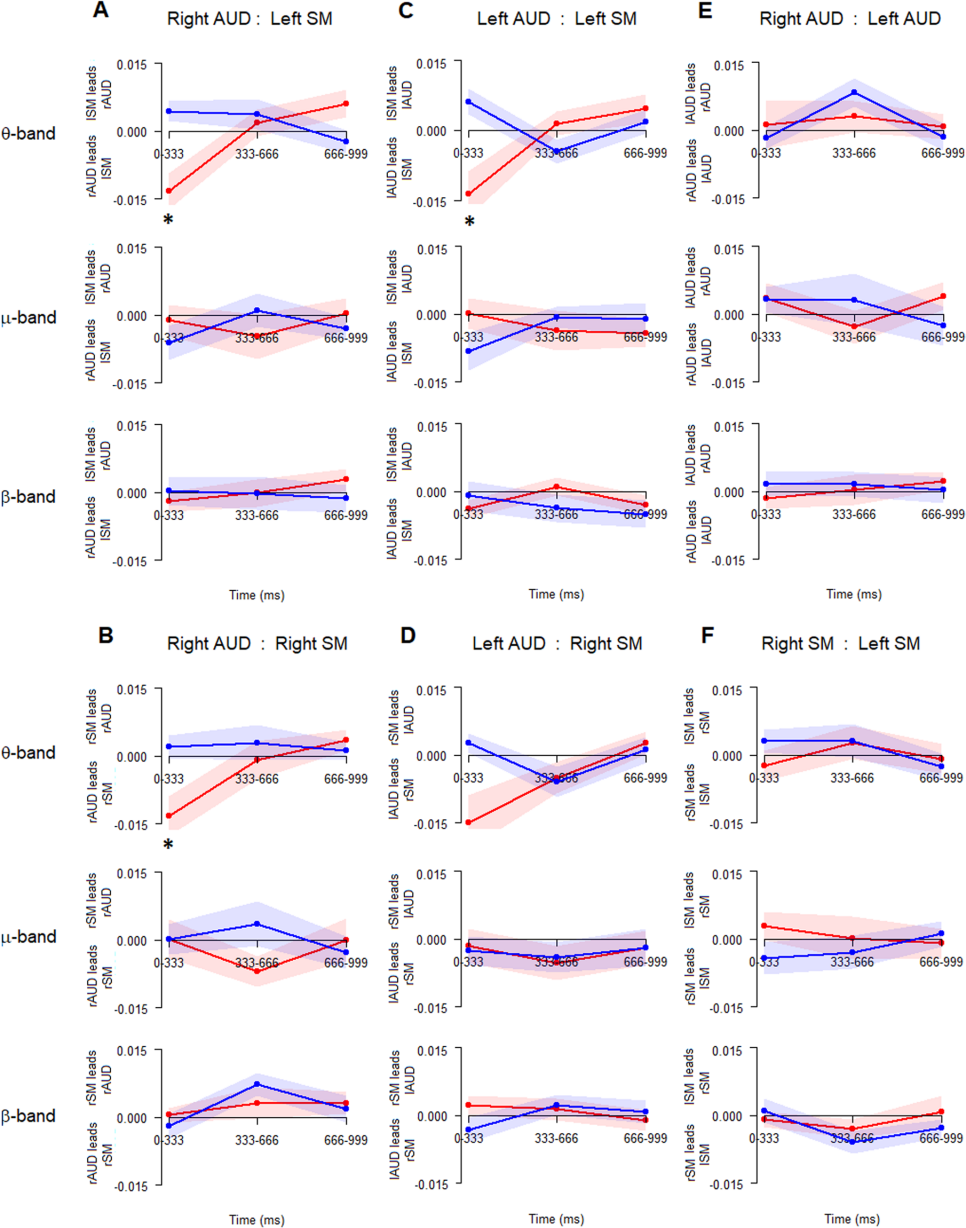
dPTE mean values for the three different frequency bands of interest, θ (1^st^, 4^th^ row), μ (2^nd^, 5^th^ row), and β (3^rd^, 6^th^ row) during Perception (red) and Imagery (blue) for all connections (via column): (**A**) rAUD to lSM; **(B)** rAUD to rSM; **(C)** lAUD to lSM; **(D)** lAUD to rSM; **(E)** rAUD to lAUD; **(F)** rSM to lSM. Values were calculated over three equal epochs division of the 1 second arrow presentation. Shaded regions are the standard error of the mean. (**A**–**D**) positive values indicate the sensorimotor region activity leading the auditory region (top-down), negative values indicate the auditory region activity leading the sensorimotor region (bottom-up). (**E**,**F**) positive values indicate the left source activity leading the right source, negative values indicate the right source activity leading the left source. * p < 0.005 FDR corrected.

Directed phase transfer entropy (dPTE) is a method that has recently been applied to measuring the effective connectivity between regions of interest^35,41,42^. The time course for each of the last 3 arrows was epoched into 3 equal segments (0–333 ms, 333–666 ms and 666–999 ms from onset of arrow) and band pass filtered into three frequency bands of interest θ (4–8 Hz), μ (8–12 Hz) and β (15–25 Hz), for each condition. (*Methods*). Imagery and Perception dPTE values revealed several significant differences after FDR correction for the θ-band only. The mean and standard error of the dPTE values for Imagery and Perception are plotted for the right auditory source connections (Fig. 7A,B), the left auditory source connections (Fig. 7C,D) and for the hemisphere connections across same sources (Fig. 7E,F). At the onset of the arrow and tone in the Perception condition (0–333 ms epoch), in the θ-band, the bilateral auditory regions (rAUD, lAUD) led the sensorimotor regions (rSM, lSM) more than during the Imagery condition (rAUD to lSM: *t*(18) = −3.575, *p* = 0.0194; rAUD to rSMr: *t*(18) = −3.175, *p* = 0.0315; lAUD to lSM: t(18) = −3.772, *p* = 0.0195, FDR corrected). In order to confirm that Imagery showed an opposite direction of information flow, and not just a difference in information flow, t-tests were conducted against 0 for each of these epoch/connections. Only the left sensorimotor to left auditory region connection showed a dPTE value that was significantly above zero (rAUD to lSM: *t*(18) = 1.971, *p* = 0.064; rAUD to rSM: *t*(18) = 0.786, *p* = 0.442; lAUD to lSM: t(18) = 2.259, *p* = 0.0365). This suggests that at the onset of the arrow, when a tone was heard (perception) information flowed from left auditory to left sensorimotor, however the opposite direction of information flow occurred when the tone was imagined. Interestingly, this pattern is reversed during the last epoch (666–999 ms), for the left sensorimotor to right auditory connection, where the left sensorimotor region leads the auditory in the Perception condition, but the right auditory leads the left sensorimotor in the Imagery. However, this difference was not significant after FDR correction (p = 0.051).

During the middle epoch (666–999 ms), when the image of the tone was being generated or updated, following the visual perception of the arrow, the left auditory regions leads the θ-band connectivity to all other regions (Fig. 7[C,E] (1^st^ row); D (4^th^ row)), whilst the left sensorimotor regions also leads both the right auditory region (Fig. 7A(1^st^ row)) and the right sensorimotor region (Fig. 7F, (4th row)). No significant difference between Imagery and Perception were found in the dPTE in μ-band or β-band between any connections.

Taken together these results show that in the Perception condition, information flow in the θ-band is directed from the auditory to the sensorimotor regions at the onset of sound; however, the (left) sensorimotor cortex drives the bilateral auditory regions during the anticipation of a sound. The latter pattern, is also seen at the onset of silent arrows for Imagery condition, suggesting the invocation of motorically-driven predictive mechanisms. Further, the left auditory source driving the bilateral sensorimotor and the right auditory activity, along with the left sensorimotor source also driving both right hemisphere sources, during the middle epoch of Imagery, supports an important role for the left hemisphere in pitch memory^43,44^ and in pitch manipulation^38^.

### Regression modelling

**Figure 8:**
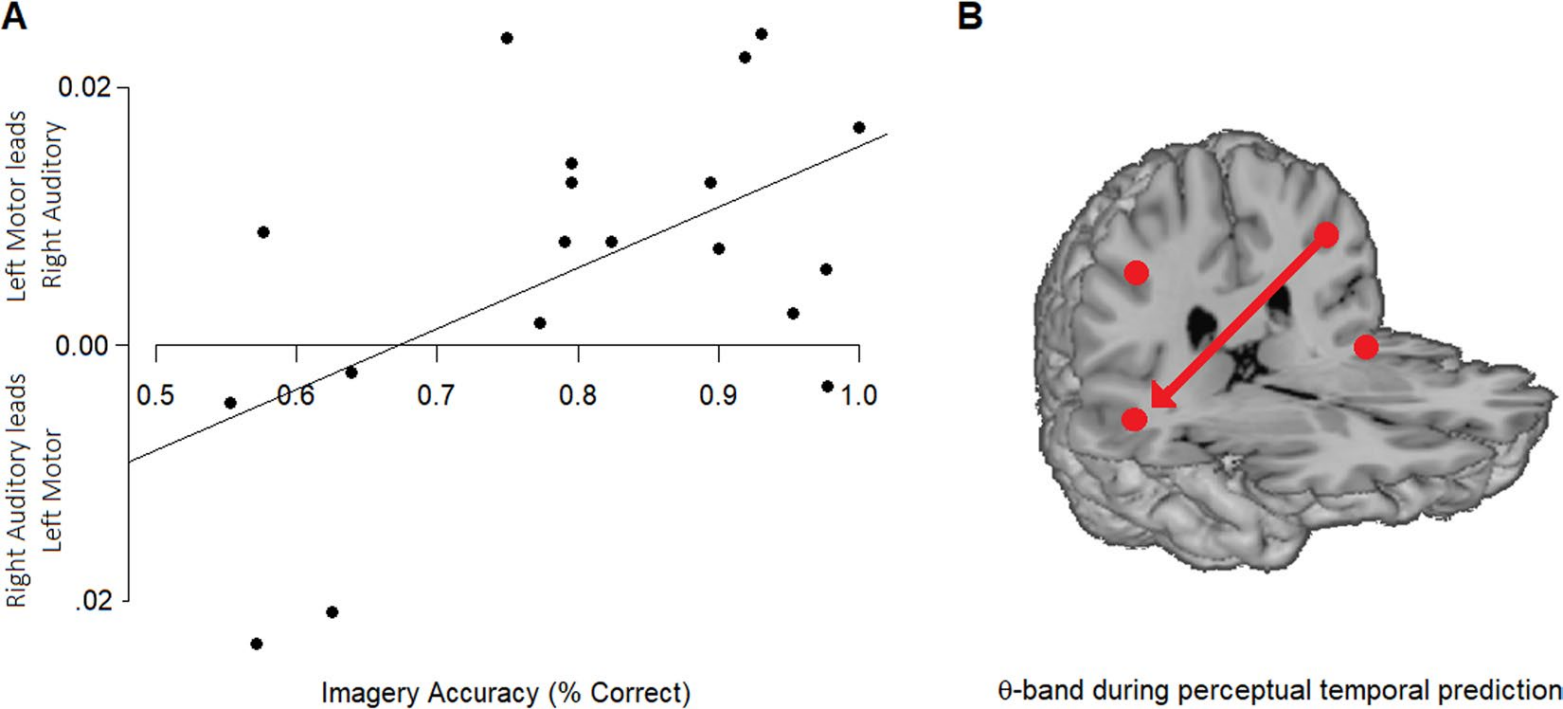
(**A**) dPTE for the right auditory/left sensorimotor connection in the Perception condition in the time window just prior to the next arrow (666–999 ms). (**B**) Schematic of directed connectivity between left sensorimotor and right auditory during this period. Imagery accuracy is positively associated with greater top-down directed connectivity from the left sensorimotor to the right auditory source.

The above results show that in the θ-band, the left sensorimotor cortex led the right auditory region in the last epoch in the Perception condition. Since it has been posited that temporal prediction in perception may require imagery^18^, we explored the nature of this relationship further through regression modelling to determine if imagery ability is facilitated by sensorimotor processing that precedes auditory processing. Linear regression was calculated using the dPTE value of the right auditory to the left sensorimotor connection in Perception condition in the last epoch (666–999 ms) as the criterion variable. The predictor variable in the initial regression was Imagery accuracy (percent correct Imagery condition) alone. The result was significant (*F*_(1, 17)_ = 6.951, *p =* 0.017), with Imagery accuracy alone accounting for 29% of the variance in dPTE value (R^2^ = 0.290; R^2^_adj_ = 0.248) (see Fig. 8). The addition of BAIS total scores or Imagery reaction time made no significant improvements to the model.

## Discussion

The results of this investigation support hypothesis (1): greater β-band modulation occurred during pitch imagery than pitch perception or maths. Whilst the β-band has previously been shown to be involved in predictive timing of ‘when’ events will occur in both perception^26^ and imagery^25^, only recently has evidence emerged that it is also important in perception for predicting ‘what’ events they may be^29,30^. For the first time, our study shows that even in silence, while *imagining* pitches, increased β-band modulation is observed, suggesting that β-band is also involved in ‘what’ is being imagined. Contrary to hypothesis (2), our analyses showed no significant hemispheric in β-band modulation for any type of cognitive operation. In relation to hypothesis (3), there was no evidence for a differential connectivity during perception and imagery in the β-band or μ -band. However, in the θ-band greater auditory to sensorimotor connectivity was seen during perception than imagery, and imagery ability was related to increased sensorimotor to auditory directed connectivity in perceptual temporal prediction. Our results may have implications for our understanding of how sensorimotor brain regions interact to support musical imagery, and how imagery-like processes may support perception.

In any study of a subjective phenomenon such as mental imagery, it is necessary to ensure that the activity of interest is taking place. Our results support the assertion that participants were using pitch imagery to complete the task. Firstly, objective behavioural measures of accuracy and reaction time measured performance and were significantly correlated with an independent measure of individual imagery ability (BAIS). Secondly, all participants confirmed the use of a musical imagery strategy to complete the task (i.e. hearing the sounds in their head or singing them in their head).

Greater β-band power modulation was seen during pitch Imagery than the Perception condition, as evidenced both in the initial normalised timecourse plotting (Fig. 4) and in the 1 Hz power calculations (Fig. 5F). The follow up analysis of modulation metrics confirmed that Imagery showed significantly greater Magnitude Difference than both Perception and Maths. This suggests that pitch imagery, completed in silence, not only significantly modulates the β-band more than perception, but also that this modulation is specific to pitch imagery (and not other silent manipulations such as mental arithmetic).

The dynamics of the β-band in response to sound has been argued to contain both exogenous components, seen in the initial β-ERD, and endogenous components, seen in the subsequent rebound, β-ERS^36^. Much attention has been given to latter, suggesting a role of the β-band rebound in predictive timing and rhythm perception over intervals of less than 1 second^24,26,45^, with the same predictive effect not seen in longer inter stimulus intervals^46^. In addition, auditory β-band activity tracks the location in time of imagined beats during a presentation of a syncopated rhythm^47^.

In this task the ‘when’ of arrow presentation was kept constant, while the ‘what’ varied (pitch to be imagined or heard, or a mathematical operation). Hence the emphasis during imagery was on pitch manipulations, and not the predictive timing of events. This is due to the arrows providing a visual isochronous metronome at a rate of 1 per second which minimised the anticipatory timing^48^ and was a slower one than the human preferred tempo^28^. Flashing visual metronomes are much less effective for synchronising than auditory metronomes at the same tempos^49^ and do not induce a strong sense of beat^50^.

Whilst β-band dynamics have been extensively studied in the role of anticipation of auditory beats, recently the role of β-band in predicting not only ‘when’ but ‘what’ a pitch will be, have been explored^29,30^. Chang *et al*.^29^ used a mismatch negativity (MMN) paradigm, with either 10% or 20% deviant tones and two different presentation rates (500 ms, 610 ms). Discrete Fourier Transforms were calculated for the resulting β-band time courses and peaks were found at the presentation rate of 2 Hz and 1.6 Hz respectively. They use this as evidence that the β-band entrains to the different inter-onset intervals in isochronous sequences^29^. Though not compared statistically, the left auditory 2 Hz power in the 20% deviant appears larger than the 10% deviant condition, suggesting greater entrainment when a deviant is more predictable. This is confirmed in a follow-up study, that showed greater β-band modulation was seen prior to predictable than un-predictable tones^30^. Further, a trial-by-trial analysis revealed that greater β-ERD prior to a predictable deviant tone was related to reduced P3a amplitude after that tone, suggesting a reduced attention-prediction error response^30^. Our study has shown greater modulation of the β-band during pitch imagery in a task that crucially has constant presentation rate across all conditions, thereby minimising uncertainty about timing. This confirms that β-band is involved not only in ‘when’ but also ‘what’ is to occur. Even in the absence of a sound, the internal generation or manipulation of a sound image corresponded to greater modulation of β-band than for perception.

Our neurophysiological measures were analysed to provide sufficient spatial sensitivity to discriminate broadly between neural activity in the two hemispheres, and between auditory and sensorimotor regions within hemispheres. Within each of the three conditions, similar time courses were seen between the auditory and sensorimotor sources in the β-band. Evidence for this is seen in the finding of no source or hemisphere differences in 1 Hz power in the β-band time series (Fig. 5E). In addition, no differences in the timing or amount of Maximum β-ERD or β-ERS were seen between sources or hemispheres. The β-band has historically been viewed as a sensorimotor rhythm^22,23,51^. Our results are consistent with the concept of β-band as an “open line” of communication between the regions, as predicted by forward and inverse feedback loops between the regions^19^. This open line of communication appears to be present during perception, imagery as well as mental arithmetic.

Our connectivity analysis revealed that the left sensorimotor cortex leads activity in the auditory regions early in the imagery onset, and the opposite directionality holds for perception. Interestingly, these directions are reversed in the latter part of the trials. Further, accuracy of imagery predicted a significant portion of variance in the connectivity in the perception condition. This finding is relevant to the suggestions by Moore^17^ and Schaefer^18^ who posited two different types of imagery: an obligatory form that is invoked to aid perception (“constructive imagery”); and a form of “sensory imagery” that can be invoked voluntarily in the absence of sensory input. To our knowledge, this is the first empirical evidence for this proposition.

In addition, our connectivity results suggest that the left hemisphere plays an important role in pitch imagery during the mid-portion of the imagery trial. While the right hemisphere has been repeatedly implicated in musical imagery^39,52^, most previous studies have not isolated pitch imagery from other aspects of the musical image (i.e. rhythm, melody). The present results are however consistent with those of brain stimulation studies, showing a causal role in pitch memory for the left but not the right supramarginal gyrus^43,44,53^. Further, θ-frequency stimulation to the left intraparietal sulcus has been shown to boost performance for mental manipulation of melodies^38^.

Finally, we found no evidence for any regional patterning of β-band connectivity during imagery or perception. For all connections, the normalised dPTE values in both conditions remained around 0. This finding stands in contrast to the results of Morillon and Baillet^35^, who reported top-down directed connectivity during temporal prediction of a melody. In the present study, this top-down pattern was observed in the θ-band. However, given the sample of participants contains a majority of musicians it could be that musicians rely more on motor regions during imagery than non-musicians. Future studies could address whether a sample of only non-musicians would show the same patterns of activity.

In summary, this is the first study to show that β-band sensorimotor oscillations are modulated more by pitch imagery manipulation than by perception, and the first to show oppositely-directed θ-band connectivity in imagery and perception, particularly in the left hemisphere. Further, our data provide evidence that imagery performance is associated with the *degree* of connectivity directed from sensorimotor to auditory regions during temporal prediction in perception.

## Materials and Methods

### Participants

64 participants were trained and screened for imagery performance in a separate behavioural testing session. We selected those who obtained a score of greater than 70% in imagining 3 successive pitch transformations in the Pitch Imagery Arrow Task (PIAT)^40^, and who reported using musical imagery to complete the task. 31 participants met the screening criteria and of these 19 participants (14 females, mean age = 25 years; range: 18–49 years) were recruited for the MEG study. All participants provided written informed consent and all procedures were approved by and performed in accordance with the relevant guidelines and regulations of the Macquarie University Human Research Ethics Committee. Participants ranged in musical training from novice (no formal training) to professional musicians. On average, participants had spent 63% of their life’s years actively engaging in musical activities, and 2 out of 19 had negligible musical training.

### Stimuli and apparatus

Presentation® software (Version 18.0, Neurobehavioral Systems, Inc., Berkeley, CA) was used to control the experiment and to record responses. Acoustic stimuli were generated from the ‘Piano’ instrument sound by Finale 2012 software (Makemusic Inc; Eden Prairie, MN) and exported as .wav files for use in Presentation®.

### Imagery screening task

The Pitch Imagery Arrow Task (PIAT) was designed to reliably induce pitch imagery in individuals with a range of musical training^40^, and was combined with two control conditions: Perception and Mental Arithmetic (Maths). Musical trials began with an ascending major scale to provide a tonal context. A start note (either tonic or dominant of scale) was then presented simultaneously with the visual presentation of a dot on the screen. A variable number, between 3–5, of up/down arrows was next displayed in random order, with each arrow accompanied by a corresponding pitch that moved up/down the scale in stepwise motion. For Imagery trials participants were required to imagine pitch steps indicated by the arrow direction, when arrows were presented in silence. A pre-probe screen appeared 1 second before an audible probe pitch, and participants indicated whether the probe matched the final imagined tone, or in the case of Perception trials, then last tone heard. The probe was correct 50% of the time, and when incorrect was within the key signature and a maximum of 2 steps away from correct answer. Participants did not know if they were performing a perception or imagery trial until the pre-probe screen appeared.

Maths trials began with the instruction “Begin Mental Arithmetic” in silence. A starting number then displayed on the screen, followed by a series of the same visual presentation of arrows, but this time in silence, with numbers above/below the point of the arrows corresponding to addition (up arrow) and subtraction (down arrow) of an ongoing mental calculation. The final probe was a visual number that corresponded to a correct or incorrect answer to this calculation. For more details see^40^.

### MEG task

The PIAT was modified in several respects for the MEG study. All Imagery trials required 3 imagined tones (rather than variable, ranging from 1–5), all conditions had an equal number of trials (n = 80 trials) and were randomly interleaved rather than blocked. No feedback was provided to participants during the task. Figure 1 shows a schematic of an Imagery trial. (See Supplementary Section for schematics of Perception and Mental Arithmetic trials). MEG data were analysed for epochs corresponding to the final three arrows cues.

### Bucknell auditory imagery scale

Participants completed the Bucknell Auditory Imagery Scale BAIS^54^. The 7-point Likert scale includes two sub scales, for vividness (BAIS-V) and control (BAIS-C). The scale items have high reliability (BAIS-V: α = 0.83, BAIS-C: α = 0.81, total scale: α = 0.91)^54^.

### Data acquisition

Brain activity was recorded with a whole-head MEG system (Model PQ1160R-N2, KIT, Kanazawa, Japan) consisting of 160 coaxial first-order gradiometers with a 50 mm baseline. Prior to MEG measurements, five marker coils were placed on the participant’s head and their positions and the participant’s head shape were measured with a pen digitizer (Polhemus Fastrack, Colchester, VT). Head position was measured by energizing the marker coils in the MEG immediately before and after each block within the recording session. During acquisition MEG was sampled at 1 kHz and band-pass filtered between 0.03 and 200 Hz. Individual structural magnetic resonance images were not available for the present experiment so the adult template brain in BESA Research 6.1 (BESA Research, Grafelfing, Germany) was used for all participants, using a spherical head model.

There were 80 trials in each of the three conditions. Approximately every 15 trials participants received rest time consisting of a blank screen for several seconds. Participants were asked to remain as still as possible and to limit eye blinks to the inter trial period or beginning of each trial. Button box (Current Designs Inc: Philadelphia, USA, Model HHSC-2 × 2) responses were made with the right index finger. Practice trials with feedback were provided prior to the start of data acquisition. The average time taken to complete the task in the MEG was approximately 56 minutes.

Surface electromyography (EMG) using BrainAmp ExG MR 16 P (BrainProducts Gmbh, Gilching, Germany) was also measured using two pairs of bipolar electrodes attached to the orbicularis oris and laryngeal muscle. EMG was viewed in real-time during the experiment to rule out any systematic muscle activities during the tasks that would indicate vocalization of the imagined notes or mental arithmetic.

### Source localisation

MEG analyses were carried out in BESA Research 6.1 (BESA Research, Gräfelfing, Germany). Our spatial filtering approach used four dipole sources modelled in bilateral auditory cortices and bilateral sensorimotor cortices. Auditory sources were fit to each participant’s data using the rising half of the averaged M100 response to the onset of each (sounded) arrow in both Imagery and Perception trials (~400 trials). Locations of sensorimotor sources were obtained from beamformer analysis of the β-band ERD to all button press responses (240 trials) for each individual participant. Bilateral sources were obtained for 17 out of 19 participants, while for 2 participants the right sensorimotor source was taken as the mirror opposite of the left. The auditory and sensorimotor sources were then combined into a single model for each participant and time frequency analysis was conducted for each source, and orientations of the sensorimotor sources were optimised in BESA Research 6.1.

Source modelling reduced the MEG data to a 4-source montage and provided spatial filters in close spatial proximity to bilateral auditory and sensorimotor cortices (Fig. 2). Subsequent analyses were computed using the 4-source montage and using data only from trials with correct responses.

Average Talairach coordinates of the auditory sources were x = 52.3 (right), y = −21.1 (posterior), and z = −1.6 (superior) in the right hemisphere (Right Superior Temporal Gyrus, BA = 21) and x = −52.1, y = −23.3, and z = 2.3 in the left hemisphere (Left Superior Temporal Gyrus, BA = 22). Talairach coordinates for sensorimotor sources were: x = 38.5 (right), y = −25.4 (posterior), and z = 45.4 (superior) in the right hemisphere (Right Postcentral Gyrus, BA = 3) and x = −40, y = −29.4, and z = 47.6 in the left hemisphere (Left Postcentral Gyrus, BA = 40).

A 5 second analysis epoch was defined with t = 0 s aligning with the onset of the first of the last three arrow cues in the trial. Hence the epoch consisted of: the last three arrow presentations (0–3 s), the retention period (3–4 s) and the probe and response period (4–5 s). The baseline for each trial was defined as the last 900 ms of the inter-trial interval before the trial. Trials with MEG artefacts including blinks and eye-movements during 0–5000 ms were rejected from the time-frequency calculations using the artefact scan tool in BESA Research 6.1.

Time-frequency plots were generated with a frequency range of 2 to 80 Hz, a frequency sampling of 1 Hz and a time sampling of 50 ms. The plots show the amplitude for each time point normalised to the mean amplitude of the baseline epoch for that frequency. A value of the time-frequency plot describes the spectral change of activity at time *t*, relative to the activity during the baseline epoch. The evoked (averaged) signal was subtracted from all trials prior to computing the mean time-frequency transform. Average time courses for the evoked response (3–10 Hz), induced β-band (15–25 Hz), and induced mu (μ) band (8–12 Hz) were calculated in MATLAB 8.2 (MathWorks Inc, MA, United States).

### 1 Hz power modulation

The 1 Hz power of during the last three arrows (0–2.95 secs) for each condition and source, were calculated using a Fast Fourier Transform, that was zero padded with a frequency resolution of 0.2 Hz. ANOVAs then compared the 1 Hz Power in the three oscillatory bands of interest (Evoked Response, μ-band and β-band) across conditions and sources. To further investigate this modulation, the Maximum ERD and ERS in the β-band, relative to the arrow cue, was calculated. The timing and amount of Maximum ERD was measured at 50–450 ms after each of the last three arrow cues, and averaged. Similarly, the timing and amount of Maximum ERS was measured between 500–950 ms after each of the last three arrows, and averaged. The difference in both timing and amount of Maximum ERS and ERD were also calculated per arrow, and averaged, to measure the range of modulation as the average time from maximum desynchronisation to reach maximum rebound, as well as the range of amplitude change over this time. ANOVAs were then conducted (3 Conditions (Imagery; Perception; Maths) x 2 Sources (Auditory; Sensorimotor) x 2 Hemispheres (Right; Left)) for timing and amount of Maximum ERD and ERS, as well as difference in timing and amount between Maximum ERD and Maximum ERS, separately for β-band. Whenever Mauchley’s test of sphericity was significant a Greenhouse-Geisser correction was made. Multiple comparisons were also controlled for using False Discovery Rate^55^.

### Directed phase transfer entropy connectivity

This directed functional connectivity method calculates the instantaneous phase of the time series from each region of interest, and, like Granger causality, determines the direction of information based on temporal precedence and influence of one region on another^41^. The advantage of this type of connectivity analysis is that it is model free, computationally straightforward and robust for various time windows and trial numbers^41^. To calculate dPTE values, data for each participant was split into 3 equal epochs across the 1 second interval between arrow cues (i.e. 0–333 ms, 333–666 ms and 666–999 ms). Epochs were concatenated, and band pass filtered into three frequency bands of interest θ (4–8 Hz), μ (8–12 Hz) and β (15–25 Hz). Using epochs of greater than 250 ms ensures that the subsequent concatenation of epochs, and then filtering, is not impacted by edge effects of concatenation in the 4–8 Hz range. Theta was chosen due to evidence of increased phase connectivity in this frequency band^36^. dTPE was calculated using *PhaseTE_MF.m*^56^, using the bin size (*h*) of Scott^57^: *h* = 3.49σ*n*^−1/3^ where σ is the standard deviation of the phase data calculated from the Hilbert transform for the time series, and *n* is the length (number of samples) of the time series. The default time delay was used, as determined by the frequency content of the data. The results were then normalised between −0.5 and 0.5 with the sign indicating the direction of the connectivity, as per Morillon and Baillet^35^. dPTE values for Imagery and Perception for each connection and each of the three epochs were then compared in t-tests (FDR corrected), to identify significant epochs and connections.

## Supplementary information

Supplementary Information

## Data Availability

The datasets collected and/or analysed during the current study are available from the corresponding author on reasonable request.
