# Supplementary material for: Musical imagery depends upon coordination of auditory and sensorimotor brain activity
Source: Sci Rep. 2019 Nov 14;9:16823. doi: 10.1038/s41598-019-53260-9 (PMC6856354; doi:10.1038/s41598-019-53260-9)
Supplement: Supplementary file 1 — Supplementary Information [file 41598_2019_53260_MOESM1_ESM.docx]

**Supplementary Information for:**

**Title:** Musical imagery depends upon coordination of auditory and sensorimotor brain activity

**Authors:** Rebecca W. Gelding *^a^, William F. Thompson ^b^, Blake W. Johnson ^a^

^a^ Department of Cognitive Science,

Macquarie University, NSW 2109, Australia

^b^ Department of Psychology,

Macquarie University, NSW 2109, Australia


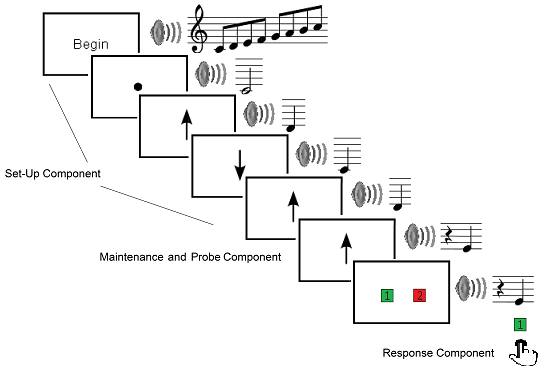


**Figure S.1.** Schematic of a MEG perception trial. Arrow cues were presented at a constant rate of 1 per second. In this example the accurate response is “Correct” which is indicated by pressing the green button.


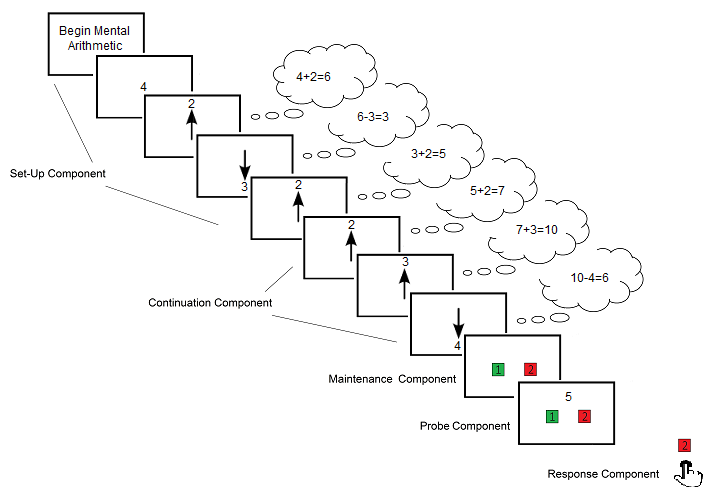


**Figure S.1.** Schematic of a MEG mental arithmetic trial. Arrow cues were presented at a constant rate of 1 per second. In this example the accurate response is “Incorrect” which is indicated by pressing the red button.
